# Enriching laying hens eggs by feeding diets with different fatty acid composition and antioxidants

**DOI:** 10.1038/s41598-021-00343-1

**Published:** 2021-10-19

**Authors:** Petru Alexandru Vlaicu, Tatiana Dumitra Panaite, Raluca Paula Turcu

**Affiliations:** Department of Chemistry and Animal Nutrition Physiology, National Research and Development Institute for Animal Biology and Nutrition, Balotesti, Romania

**Keywords:** Nutrition, Public health, Plant sciences, Risk factors, Chemistry, Engineering

## Abstract

The current study was conducted to evaluate egg quality, egg yolk fatty acids, health-related indices and antioxidants from laying hens' eggs fed different combined vegetable by-products, rich in fatty acids and antioxidants. One hundred twenty 50 weeks-old Tetra SL laying hens were divided into three groups. They were given daily a standard diet (Control, C), a diet containing 9% rapeseed meal with 3% grapeseed meal (T1 diet), or a diet containing 9% flaxseed meal and 3% sea buckthorn meal (T2 diet). Hen production performances, egg quality, egg yolk fatty acids total polyphenols content and antioxidant capacity were determined. The T1 diet significantly reduced the egg yolk content of palmitic acid from 76.615 mg (C) to 46.843 mg (T1) and that of oleic acid from 788.13 mg (C) to 682.83 mg (T1). Feeding flaxseed and sea buckthorn meals significantly increased the egg yolk content of α-linolenic acid in T2 yolks (35.297 mg) compared with C yolks (4.752 mg) and that of docosahexaenoic acid (DHA) from 16.282 mg (C) to 74.918 mg (T2). The atherogenicity indices (AI) were not significantly affected, whereas the thrombogenicity indices (TI) decreased significantly (p < 0.0007) from 0.72 (C) to 0.60 (T1) and 0.66 (T2), respectively. Adding this combination of meals to the hens' diets, increased the total polyphenol content and antioxidant capacity in T1 and T2 eggs compared to C eggs. The significant enrichment of eggs with n-3 fatty acids and antioxidant capacity, as well on the health-related indices especially from T2 eggs, represents a potential functional feed ingredient in poultry feeding, to obtain eggs as functional food.

## Introduction

Chicken eggs for human consumption are exceedingly nutritious, palatable, reasonable priced, and commonly accessible around the world. There is an increasing demand for enriched and functional foods for human consumption that provide various beneficial effects in addition to the nutritive and non-nutritive compounds important to human health^1^. Eggs can be enriched with certain nutrients through dietary manipulation to create specialty or functional food products that provide health benefits for humans^2,3^. Due to the benefits associated with the consumption of n-3 fatty acids (FA), many researchers have conducted studies in the last decades to enrich different foods of animal origin, such as broiler meat or eggs, in FA while delaying the lipid oxidation. The role of essential FA (linoleic and α-linolenic) and their long-chain (LC) n-6 and n-3 polyunsaturated fatty acids (PUFA) metabolites in human health, growth, and development is a topic of continued interest^4,5^. Among many important healthy beneficial effects, such as decreasing risk of cardiovascular disease, prevention and treatment of inflammatory diseases are some of the attributed given by the consumption of n-3 PUFA enriched eggs^6^. This type of eggs can be obtained from laying hens that are fed with different by-products rich in PUFA, like flaxseed, rapeseed, microalgae, canola, chia (seed, meals or oils), or full fat mixtures^2,7^. Previous studies concluded that the FA composition of eggs is dependent on the FA composition of the feed given to the hens^8^ which are subsequent transferred into the eggs. Fatty acids and antioxidants in eggs are essential components from a wellbeing and consumption perspective for humans, especially in terms of n-3 PUFA consumption^9^. Consequently, higher fat content incorporated into laying hens' diet through rapeseed (*Brassica rappa*) and flaxseed (*Linum usitatissimum*) meal by-products is a good alternative for PUFA eggs enrichment. However, this enrichment leads to an increment within the unsaturation of eggs, and increase the susceptibility to lipid oxidation^10^, or give unpleasant organoleptic characteristics (fishy smell) when used in higher doses. Lipid oxidation in foods is of major significance since it antagonistically influences the general quality of foods, and nutritional value^11^. In order to avoid such undesirable effects, a normal way is to extend the intrinsic antioxidant concentration through dietary incorporation of natural antioxidant, such as grape seed (*Vitis Vinifera*) or sea buckthorn (*Hippophae rhamnoides*) meals. Grape is one of the world's largest fruit crops^6^. Grape seed is a natural agricultural by-product of grapes, considered a natural source of anti-oxidative constituents (vitamin E, flavonoids, pro-anthocyanidins and polyphenols)^12^. Sea buckthorn is widely distributed all over the world. It contains different nutrients and bioactive substances such as vitamins, carotenoids, flavonoids, polyphenols and PUFA^13^. Both grapeseed and sea buckthorn meal are underexploited by-products, which have gain attention in the last years, as natural antioxidant sources with biological benefits. Dietary supplementation with natural antioxidant sources has been demonstrated to be very successful in improving poultry products in antioxidants and concomitant delay the lipid oxidation of products which could be produced by the high fat content of the egg^14^.

To our knowledge, there are little studies performed following similar dietary inclusion. Thus, the objective of the present study was to evaluate the effect of the dietary incorporation of rapeseed and flaxseed meals as different natural sources of polyunsaturated fatty acids together with a natural source of antioxidant, namely grapeseed meal and sea buckthorn meal on laying hens’ performances, egg yolk fatty acids, health-related indices, total polyphenol content and antioxidant capacity of eggs.

## Results

### Laying hens performances and egg physical quality characteristics

The usage of T1 and T2 diets had no effect (p > 0.05) on laying hens’ performances (Table 1). It was observed only a tendency of increasing the average daily feed intake and a decrease of feed conversion ratio in T2 group. Egg weight and laying rate were higher in experimental treatments, but without a notable (p > 0.05) effect. Overall, the diets used were not significant (p > 0.05) contributors in relationship to the obtained performances. The tested diets did not influence the egg weight or egg constituents (albumen, yolk and shell) (Table 1). However, egg albumen pH was significantly lower (p = 0.0245) in T2 compared with C, while the egg yolk pH was significantly lower (p = 0.0004) in both T1 and T2 compared with C samples. The yolk colour from T1 and T2 groups, measured with a Roche colour fan, was significantly (p = 0.0155) improved compared with C samples. Haugh unit (HU), parameter was 4.31% higher in T1 and 4.47% higher in T2 compared with C eggs.

**Table 1:** Effect of dietary by-products on laying performances and egg quality characteristics.

Items	C	T1	T2	SEM	p
**Laying performances**					
Average daily feed intake (g/day/hen)	118.19	118.99	119.31	0.554	0.1741
Feed conversion ratio (kg CF/kg egg)	2.08	2.06	2.05	0.711	0.4340
Laying rate (%)	89.30	90.56	90.26	0.021	0.0787
**Egg quality characteristics**					
Egg weight (g)	63.50	63.53	63.72	0.156	0.8261
Albumen (g)	37.86	38.29	37.95	0.226	0.7271
Yolk (g)	17.00	16.71	16.86	0.201	0.6251
Shell (g)	8.64	8.71	8.91	0.115	0.6217
Shell thickness (μm)	350.0	350.0	360.1	0.003	0.5754
Shell strength (kgF)	3.77	4.00	3.66	0.111	0.4515
Albumen pH	8.95^a^	8.84^ab^	8.71^b^	0.036	0.0245
Yolk pH	6.23^a^	6.21^b^	6.20^b^	0.012	0.0004
Yolk colour fan	4.67^c^	5.06^b^	6.78^a^	0.082	0.0055
Haugh units (HU)	82.12	85.82	85.97	1.092	0.6169

*C* control diet, *T1* control diet with 9% rapeseed meal and 3% grapeseed meal, *T2* control diet with 9% flax meal and 3% buckthorn meal, *SEM* standard error of the mean, *p* significance.

^abc^Mean marked with a different superscript letter within each column are significantly different.

### Fatty acid composition of the analysed eggs

The effect of using diets rich in PUFA and antioxidants on the FA composition of egg yolk is shown in Table 2. Among the SFA, the most abundant were myristic (C14:0), which has been significantly (p < 0.0051) higher in C compared with T1 and T2. Additionally, palmitic acid (C16:0) was significantly (p < 0.0001) higher in C and T2 compared with T1 egg yolks, followed by stearic (C18:0) which had the tendency to be higher in T2 compared with C and T1. Heptadecanoic acid (C17:0) was significantly (p < 0.0603) higher only in T1 compared with C egg yolks. Regarding the MUFA content in egg yolk samples, palmitoleic acid (C16:1) and oleic acid (C18:1) were significantly (p < 0.05) higher in the samples from C and T2 compared to T1 yolks. Nervonic acid (C24:1n9) was significantly (p < 0.0001) higher in both C and T1 compared with T2 samples. Furthermore, the birds which consumed T1 diet have deposited higher (p < 0.0001) amounts of n-6 PUFA, while the birds which consumed T2 diet have deposited higher (p < 0.0001) amounts of n-3 PUFA, as a response of different PUFA sources added in diets. From the total essential n-6 PUFA determined in egg yolks from T1, linoleic acid (C18:2n6) and arachidonic (C20:4n6) were significantly (p < 0.001) higher compared with C and T2. Nevertheless, out of the total essential n-3 PUFA, the most important and dominant ones involved in human physiology were identified in egg yolks from T2. The α-linolenic (C18:3n3) was almost eight times higher compared with C and T1 eggs. Moreover, the docosapentaenoic acid (C22:5n3) was significantly (p < 0.0001) higher, while the docosahexaenoic acid (C22:6n3) was almost five times higher compared to the concentrations determined in C and T1 egg yolks, as a response of flax meal added in this diet.

**Table 2:** Effect of dietary by-products on the egg yolk fatty acids profile.

Item	C	T1	T2	SEM	p
	mg fatty acids/yolk				
Yolk Fat, % DM	27.24	27.04	27.06	0.217	0.9250
Myristic C14:0	7.390^a^	5.505^b^	5.662^b^	0.281	0.0028
Pentadecanoic C15:0	1.518	1.575	1.693	0.072	0.6241
Palmitic C16:0	582.96^a^	510.35^b^	553.67^a^	9.495	0.0015
Heptadecanoic C17:0	2.603	3.428	2.767	0.170	0.1040
Stearic C18:0	247.20	268.57	239.50	7.495	0.2742
∑SFA	841.68	789.43	803.28	12.628	0.2254
Pentadecenoic C15:1	2.598	2.802	2.703	0.154	0.8790
Palmitoleic C16:1	76.615^a^	46.843^b^	76.402^a^	3.990	0.0005
Heptadecenoic C17:1	1.925	2.512	2.268	0.150	0.2990
Oleic C18:1	788.13^a^	682.83^b^	778.99^a^	17.838	0.0170
Erucic C22:1n9	2.536	3.068	2.086	0.255	0.2979
Nervonic C24:1n9	8.512^a^	8.653^a^	3.935^b^	0.594	< 0.0001
∑MUFA	876.79^a^	746.69^b^	867.67^a^	21.028	0.0088
Linoleic C18:2n6	425.18^a^	501.70^b^	369.86^c^	16.651	0.0007
Linolenic γ C18:3n6	2.912^a^	2.340 ^b^	2.014^b^	0.132	0.0061
Eicosadienoic C20:2n6	3.542	4.175	2.888	0.309	0.2274
Eicosatrienoic C20:3n6	6.274^b^	7.802^a^	4.678^c^	0.402	0.0005
Arachidonic C20:4n6	94.422^a^	105.238^a^	3.40^b^	0.168	0.0005
Docosatetraenoic C22:4n6	37.29^a^	35.432^a^	5.742^b^	3.640	< 0.0001
α-Linolenic C18:3n3	4.752^b^	4.355^b^	35.297^a^	3.571	< 0.0001
Eicosatrienoic C20:3n3	5.315	5.508	6.408	0.347	0.4137
Docosapentaenoic C22:5n3	1.755^b^	1.498^b^	5.60^a^	0.486	< 0.0001
Docosahexaenoic C22:6n3	16.282^b^	16.352^b^	74.918^a^	6.766	< 0.0001
∑PUFA	596.55^b^	682.72^a^	584.25^b^	16.757	0.0211
n-3 PUFA	28.103^b^	27.713^b^	122.23^a^	10.877	< 0.0001
n-6 PUFA	568.45^b^	655.05^a^	462.03^c^	22.596	< 0.0001
n-6/n-3	471.39^a^	528.25^a^	85.252^b^	48.881	< 0.0001
∑UFA	1473.34	1429.41	1451.92	21.889	0.7582

*SFA* saturated fatty acids, *MUFA* monounsaturated fatty acids, *PUFA* polyunsaturated fatty acids, *n-6* omega 6 fatty acids, *n-3* omega 3 fatty acids, *UFA* unsaturated fatty acids, *C* control diet, *T1* control diet with 9% rapeseed meal and 3% grapeseed meal, *T2* control diet with 9% flax meal and 3% buckthorn meal, *SEM* standard error of the mean, *p* significance.

^abc^Mean marked with a different superscript letter within each column are significantly different.

### Health-related lipid indices

The obtained health indices calculated in the present study are outlined in Table 3. Saturation indices (SI) and atherogenic indices (AI) were higher in C eggs, but without significant (p > 0.05) alteration in contrast with T1 and T2. The thrombogenicity indices (TI) were significantly (p = 0.0007) different among all egg samples. As expected, the peroxidability indices (PI) was significantly (p < 0.0001) higher, in T1 and T2 vs. C group. The values of oxidative susceptibility (OS), desirable fatty acids (DFA), nutritive value (NVI) and ratio between hypocholesterolemic and hypercholesterolemic (HH) health indices from T1 and T2 egg samples, presented significantly (p < 0.0001) higher values than in C eggs. In term of hypercholesterolemic saturated fatty acids (HFSA), egg samples from C group had the highest (p < 0.0001) value (25.50), followed by T2 (24.80) and T1 (23.29). On the other hand, C egg samples had the lowest oxidisability value (Cox) (p = 0.0001).

**Table 3:** Effect of dietary by-products on health-related lipid indices of egg yolks.

Items	C	T1	T2	SEM	p
Saturation indices (SI)	0.57	0.55	0.55	0.007	0.4631
Atherogenicity indices (AI)	0.58	0.56	0.56	0.007	0.3552
Thrombogenicity indices (TI)	0.72^a^	0.60^c^	0.66^b^	0.015	0.0007
Peroxidability indices (PI)	27.37^c^	30.91^b^	44.92^a^	1.919	< 0.0001
Hypo/hypercholesterolemic (HH)	0.81^c^	1.18^a^	0.94^b^	0.038	< 0.0001
Hypercholesterolemic saturated fatty acids (HSFA)	25.50^a^	23.29^b^	24.80^a^	0.267	< 0.0001
Oxidisability value (Cox)	2.30^b^	2.70^a^	2.39^b^	0.048	0.0001
Oxidative susceptibility (OS)	897.70^c^	1075.56^a^	979.60^b^	3.036	0.0001
Desirable fatty acids (DFA)	74.11^b^	75.98^a^	76.47^a^	0.263	0.0001
Nutritive value indices (NVI)	1.77^b^	1.86^a^	1.84^ab^	0.018	0.0088

*C* control diet, *T1* diet supplemented with 9% rapeseed meal with 3% grapeseed meal, *T2* diet supplemented with 9% flaxseed meal with 3% sea-buckthorn meal, *SEM* standard error of the mean, *p* significance.

^abc^Mean marked with a different superscript letter within each column are significantly different.

### Antioxidant compounds of the eggs

In the Table 4 are presented the effects of dietary by-products added in laying hens' diets on total polyphenol content (TPC) and total antioxidant capacity (TAC) determined in eggs. The T2 group, which was supplemented with flaxseed and sea buckthorn meals, had significantly (p < 0.001) increased the TPC compared with C. Similarly, the TPC determined in eggs from T1, supplemented with rapeseed and grapeseed meals, led to an increase of 4.03% higher than C, but without statistical (p > 0.05) differences. The eggs from T2 were also constant in terms of TAC determined in both yolk and albumen egg. The methanolic egg yolk and albumen extracts exhibited statistically significant differences in TAC, as measured using the DPPH method. The highest concentration was observed in both yolk (80.28 mM Trolox/g) and albumen (86.15 mM Trolox/g) extract from T2 eggs, followed by the extract from T1 egg (respectively 79.56 mM Trolox/g and 85.30 mM Trolox/g), which resulted being significantly (p < 0.001) higher compared with C egg yolk (75.34 mM Trolox/g) and egg albumen (80.47 mM Trolox/g).

**Table 4:** Effect of dietary by-products on antioxidant compounds determined in eggs.

Items	C	T1	T2	SEM	p
Total polyphenol concentration (TPC) (mg GAE/g egg)	40.94^b^	43.66^a^	45.36^a^	17.385	0.0075
Total antioxidant capacity TAC) (mM Trolox/g yolk)	75.34^b^	79.56^a^	80.28^a^	1.130	0.0015
Total antioxidant capacity (TAC) (mM Trolox/g albumen)	80.47^b^	85.30^a^	86.15^a^	0.946	0.0094

*C* control diet, *T1* control diet with 9% rapeseed meal and 3% grapeseed meal, *T2* control diet with 9% flaxseed meal and 3% sea buckthorn meal, *SEM* standard error of the mean, *p* significance.

^ab^Mean marked with a different superscript letter within each column are significantly different.

### The relationship between egg characteristics given by principal components analysis (PCA)

**Figure 1:**
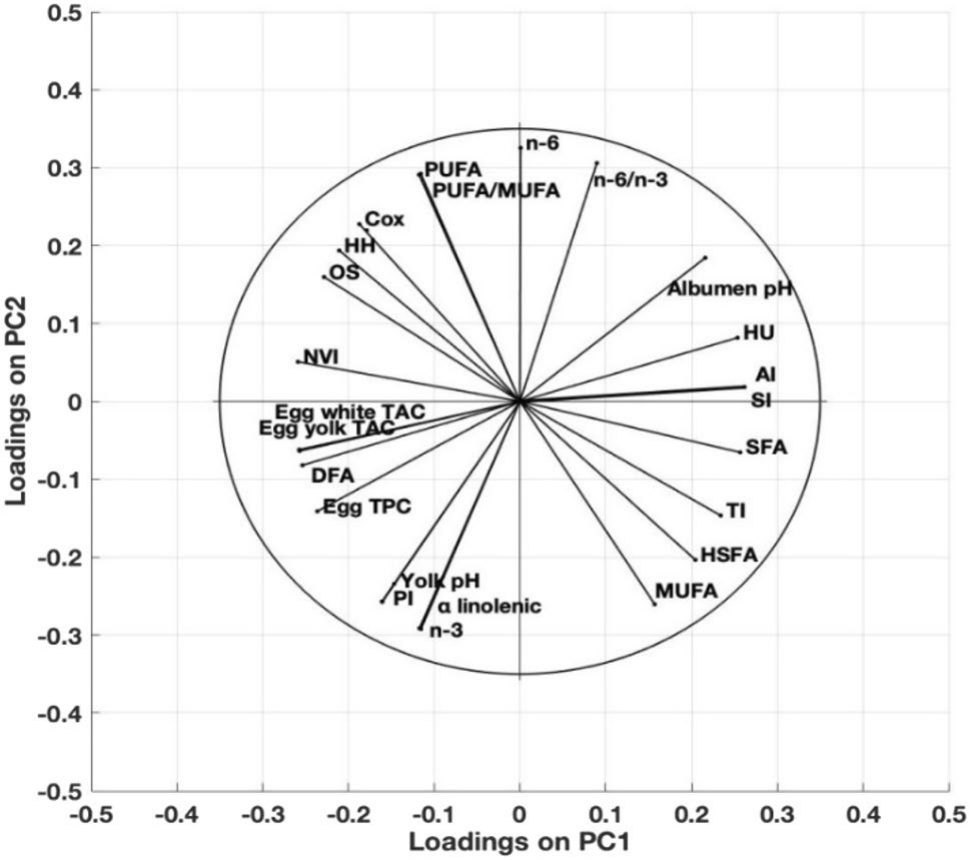
Principal component analysis and correlation loading of fatty acids, health indices, total phenolic compounds, antioxidant capacity, HU, yolk and albumen pH of the analysed egg samples.

To verify which parameters were more related with the detected differences, the PCA was applied to all determined egg quality parameters (Fig. 1). PCA is one of multivariate statistical methods used for exploratory data analysis to uncover hidden relationships between various parameters taken at once. The loadings of the original variables on the first two principal components, was described by 75% of the total variance for PC1 and by 20% for PC2. Our data showed that there is a positive correlation between health indices and antioxidant compounds, while the antioxidant compounds are negatively correlated. On the other hand, it was found a strong correlation between the antioxidant compounds and fatty acids classes, as expected.

## Discussion

Our dietary treatments were without significant effect on hens' performances. The results reported in literature on the effect of these by-products (rapeseed meal, grapeseed meal, flaxseed meal or sea buckthorn meals) on laying hens used alone or together with different supplements on performances are controversial^15–17^. Various factors have been reported to have the ability to influence differently the performances^18^, including the duration of these trials or the inclusion level of this by-products. In this study, egg weight or egg constituents (shell, yolk or albumen) were not influenced by the experimental diets. In line with our findings^18^ similar results were obtained in terms of egg constituents when hens were subjected to diets rich in PUFA from flaxseed, rapeseed or fish oil. Lower pH values in egg albumen and yolk are a wanted effect, which means that the natural antioxidants added in the diets acted against lipid oxidation products in eggs. The increased egg yolk colour from T1 and T2 groups is also a beneficial result considering that yolk colour has always been regarded as an important egg quality characteristic and as a key criterion to producers and consumers who are attracted by well-pigmented egg products. Actually, consumers tend to associate golden yellow to orange yolk with good health^19^. Similarly, Momani et al.^20^ reported that 5% sea buckthorn meal had significant effect on egg yolk colour, which supports our results obtained from T2 egg yolk colour.

Manipulating laying hens' diets by adding different by-products rich in fatty acids and antioxidant resulted in significant changes in the FA profile of eggs. Compared with eggs from group C, the lowest total SFA values were determined in eggs produced from hens fed with experimental diets (T1 and T2). Palmitic acid (C16:0) was the most abundant SFA in eggs from C group (582.96 mg FA/yolk), followed by eggs from T2 (553.67 mg FA/yolk) which is known to be the primary product of FA synthetase reactions within the tissues^21^. Once it is released from the synthetase complex, it can be esterified into complex lipids, which are further elongated to de novo stearic acid (C18:0) or desaturated to the MUFA, palmitoleic acid (C16:1), involving different enzymatic pathways in each reaction^22^. The effect of significant increase in palmitoleic (C16:1) and oleic (C18:1) from C and T2 eggs compared to T1 yolks, led to a significant increase in total MUFA from C (876.79 mg FA/yolk) and T2 (867.67 mg FA/yolk) eggs when compared with T1 eggs (746.69 mg FA/yolk). These results were unexpected taking into consideration the fact that C and T1 diets had higher concentration of MUFA compared with T2. This effect has been also con-firmed by other authors^4,23^ and it happens due to the fact that FA synthesis in animal systems produce only SFA and MUFA of the n-9 series, usually oleic acid (C18:1). Furthermore, birds that consumed T1 diet deposited higher (p < 0.0001) amounts of n-6 PUFA, especially linoleic (C18:2n6) and arachidonic acids (C20:4n6) compared with concentrations determined in C and T2. This increase in T1 eggs is related to the rapeseed meal and grapeseed meal, both having higher concentrations of n-6 compared with by-products used in T2. Similarly, Halle and Schöne^24^, measured high concentration of linoleic acid determined in yolk lipids, caused by addition of 10% rapeseed cake, which is very close to our obtained result of 501.70 mg fatty acids/yolk linoleic acid in T1 eggs. Moreover, Rowghani^25^ found that by adding 3 and 5% canola oil, the percentage of total n-3 FA compared with C egg samples significantly (p < 0.05) increased. Nevertheless, other authors^26,27^ concluded that 10% rapeseed cake or different mixtures of rapeseed, flaxseed, fish oils provide the possibility of the enrichment of yolk fat with PUFA. What is more, the laying hens that consumed T2 diet deposited higher α-linolenic acid and docosahexaenoic, resulting in an increase of the total n-3 FA and a decrease in n-6 PUFA, especially arachidonic acid when compared with eggs from C and T1. Our results are in line with previous studies on laying hens fed diets containing same by-products^24,25^. In addition, Imran et al.^28^ by using 10%, 20% and 30% extruded flax meal in hen's diet reported a significant improvement of α-linolenic and docosahexaenoic acid in egg yolk with a concomitant reduction in arachidonic acid which is similar with our obtained results from T2 eggs. This is a beneficial effect considering that generally, table eggs tend to be relatively high in n-6 FA and scarce in n-3 FA, as it was previously reported^1^. The variation of SFA, MUFA and PUFA among the groups occurs due to the conversion of one FA into another, such as stearic acid in oleic acid, but also due to the action of the enzyme in the formation and depletion of FA^18^. The differences between the two experimental groups (T1 and T2), could be caused by the decreased arachidonic acid, (as in T2 eggs) which is formed from linoleic acid through desaturation and elongation in the hen liver, where α-linolenic is metabolized to LC n-3 FA by Δ6-, Δ5-, and Δ4-desaturases and elongases^29^. Furthermore, the process of desaturation and elongation, leads to a competition for the enzymes between n-3 and n-6 FA with a preference for n-3 over n-6 PUFA^30,31^. It has been shown that saturated and trans fats inhibit the Δ6-, Δ5-pathways, limiting LC-FA concentrations, but the inclusion of antioxidants (synthetic or natural) modulates Δ6-desaturase (it is the critical enzyme in these reactions) pathway in a favourable manner and increase LC n-3 FA concentration in eggs^30^, for which it has a great affinity. By reporting the fatty acid profile as mg/egg yolk, it is very clear to observe that T2 egg provide about 110.21 mg of n-3 per egg from α-linolenic acid and docosahexaenoic acid. This concentration supplies almost half of the daily recommended dose of this important fatty acids. With respect to the T1 eggs, they supply high amounts of n-6 and scarce amount of n-3, which is similar to eggs from C group. Hence, using diets rich in n-3 FA (as T2 diet) reduces the n-6 FA of egg yolk, which represents a favourable effect for consumers. This may prove to be a key food ingredient for obtaining functional foods and establishing egg consumption for promoting better health effects in humans.

Data obtained on egg lipid components, emphasize the strong influence of FA lipid profile in eggs from T1 and T2 on health lipid indices. The PUFA together with their ratio are the FA controlling the hypocholesterolemic indices. The n-3 PUFA plays a major role for regulating the TI, whereas n-6 PUFA are dominant for the AI. Healthy animal products (eggs, meat) are characterized by lower values of AI and TI but with high value of HH indices^32^. AI and TI are vital parameters for evaluating the healthiness of lipid for human consumption, indicating the potential of providing benefits for health of cardiovascular system^33^. The recommended levels for human consumption of AI and TI should be less than 1.0 as it was also found in other studies^34^. Our results showed that AI were equal in both experimental eggs (0.56) while TI were significantly lower in T1 (0.60) and T2 (0.66) compared with C (0.72). The significant decrease of total MUFA it was also reflected in the HSFA from T1 (23.29) and T2 (24.80) vs. C (25.50) eggs, which it was reported to be very effective in lowering blood cholesterol concentration and in preventing coronary heart disease in elderly people^1^. These results are very desirable from a human health point of view. Our results are in good agreement or close to those calculated on the basis of FA profile for laying hens by other authors ^35,36^. On the other hand, according to an FAO^6^ report, PI, HH, OS, DFA and NVI should be as high as possible, considered to have eitherneutral or cholesterol-lowering effects^37^. Our PI obtained values in eggs from T1 (30.91) and T2 (44.92), were higher than those from C eggs (27.37), indicating a higher pro-health value of obtained eggs. Similarly, Batkowska et al.^38^ showed higher PI values than control group. The significantly higher value of HH health indices values obtained from T1 (1.18) and T2 (0.94) egg samples, represent a beneficial effect for human health, the higher this ratio is, the more adequate that fat is for human nutrition. Similar effects have been also attributed to the oxidative susceptibility indices, which were with 16.54% higher in T1 and with 8.36% higher in T2 eggs, from hens' fed diets rich in PUFA and antioxidants. Additionally, the highest value determined for nutritive value indices (NVI) was characteristic for both T1 (1.84) and T2 (1.84) eggs compared with C (1.77) eggs. All in all, it is difficult to compare these health indices obtained in the present study from hens fed with these natural by-products. The main reason for this conclusion is the lack of re-searches and studies related to the assessment of health indices on hens’ egg yolk following similar dietary incorporation.

The concentration of PUFA, which were incorporated into eggs by feeding T1 and T2 diets to laying hens might increase the susceptibility to oxidation of the FA, considering the fact that eggs, in particular those enriched in PUFA, are highly prone to oxidative processes^39^. It was reported that the antioxidant compounds such as polyphenols, vitamins and minerals from eggs, act synergistically with each other, providing a protective effect against eventual lipid peroxidation^40^, due to efficiency of nutrient transfer from the feed to the egg. For this reason, simultaneously enrichment of eggs with polyphenols and antioxidant compounds was suggested to decrease FA oxidation and provide a good source of dietary antioxidant^8^. The results gathered in this study indicate that the by-products added in T1 and T2 diets were effective in improving egg quality after 6 weeks of feeding by increasing the polyphenols and antioxidant capacity in eggs. Moreover, it was clear that the used by-products, exhibited high antioxidant properties by manipulation of poultry feed, which further promoted a significant enhancement in polyphenols and antioxidant compounds in laying hens' eggs of experimental group compared with control group eggs. The polyphenol concentration increased in both experimental eggs, but the significant increase was noted in T2 eggs (45.36 mg GAE/g) compared with C eggs (40.94 mg GAE/g). Also, the antioxidant capacity increased significantly in egg yolk (79.56 in T1 and 80.28 in T2 mM Trolox/g) and egg albumen (85.30 in T1 and 86.15 in T2 mM Trolox/g) in both experimental groups compared with C egg yolk (75.34 mM Trolox/g) and egg albumen (80.47 mM Trolox/g). This effect is also attributed to the major role of bioactive compounds present in the added by-products as natural source of antioxidants which are responsible for health promoting action^28^. Other authors^41,42^ reported that high concentration of polyphenols implies that the antioxidants of the by-products significantly increase because of its capacity to block free radicals. This relationship between polyphenols and antioxidants was also reported by others^43,44^. In line with our results, Karakaya et al.^45^ reported that different levels of antioxidants from grapeseed and their by-products were significantly (p < 0.05) effective in fresh eggs. The effectiveness of sea buckthorn was also studied previously, as natural source of bioactive compounds in laying hens^46,47^ on egg quality, with beneficial effects. As it has been stated before, PUFA enriched eggs simultaneously with polyphenols and antioxidant compounds help antioxidant assimilation^48^. This is a wanted effect from a consumer perspective, considering that some evidence from WHO^49^ strongly supports a contribution of polyphenols to the prevention of cancers, cardiovascular diseases, and osteoporosis and suggests a role in the prevention of neurodegenerative diseases and diabetes mellitus^50^. As it was shown polyphenols improve the status of different oxidative stress biomarkers^51^ and it is established that some polyphenols, administered as supplements or with food, do improve health status^52,53^ especially protective effects against cardiovascular diseases^54^. Overall, in the light of the findings from the present study, provide additional support that the production of eggs enriched with polyunsaturated fatty acids and antioxidant compounds, which may give poultry farmers an opportunity to be part of an emerging industry that could increase marketability by offering consumers an alternate way of obtaining these health-promoting nutrients through their diet.

The PCA analysis of bi-plot of the fatty acid classes, health indices and antioxidants were carried out because this matrix is a very complex mixture. The PCA model with two significant components, and the positive or negative relationship between them was explained by 75%, of the variance in the original parameters (PC1) and by 20% for the second component (PC2), respectively. The highest positive correlation was found among the concentrations of PUFA, PUFA/MUFA ratio, n-3 and n-6 fatty acids. The TPC from both yolk and albumen, were also strongly correlated. These results suggest that phenolic compounds, such as phenolic acids and flavonoids, may be important contributors to the antioxidant capacity. From the health-related indices AI, SI, NVI and PI had the strongest correlation. For the rest of the parameters, the correlation was moderate. We also took into consideration α-linolenic FA, as the most important one, which was strongly correlated with PI indices and n-3 FA, while for the same parameter was observed a strong negative correlation with n-6 FA. A high negative relationship was also found between SFA and PUFA.

## Methods

### Ethical considerations

The study was approved before the initiation of research, by the Ethical Commission of the National and Development Institute for Biology and Animal Nutrition (INCDBNA-IBNA), Balotesti according to the experimental protocol no. 454/23 January 2019 and in compliance with the ARRIVE guidelines.

The study complied with the principles of Romanian Law 43/2014 ordinance 28/31.08.2011, and Law 43/11.04.2014 for the handling care, and protection of animals used for experimental purposes, the European Union Council Directive 98/58/EC concerning the protection of farmed animals and Directive 2010/63/EU on the protection of animals used for scientific purposes.

### Birds, diets, and housing

The by-products used in this study were procured from an oil cold pressing producer (SC 2-EProd SRL) from Alexandria, Teleorman County, Romania. Before the usage of them in laying hens' diets, they had been shredded with an everyday MCU hammer mill (7.5 kW power), with 1 mm screen. Samples from each by-product (about 500 g) were analysed for the proximate composition (dry matter, crude protein, crude fibre and ether extract), fatty acids content, total polyphenol content and antioxidant capacity (Table 5).

**Table 5:** Proximate chemical composition and nutrient profile of dietary by-products.

Specification	Rapeseed meal	Grapeseed meal	Flaxseed meal	Sea Buckthorn meal
**Proximate analysis of nutrients (% DM)**				
Dry matter	89.25	89.91	90.86	89.36
Crude protein	33.56	13.10	31.38	11.44
Ether extract	15.07	6.44	13.14	8.92
Crude fibre	10.10	35.16	11.26	23.26
**Fatty acid content (% of total fat)**				
Palmitic (C16:0)	11.92	9.68	7.70	21.56
Stearic (C18:0)	2.73	3.56	3.07	1.80
Oleic (18:1)	41.06	21.04	18.54	30.70
Linolenic (C18:3n3)	4.42	1.33	42.93	4.84
Total SFA	16.83	13.48	11.07	23.69
Total MUFA	42.90	21.34	18.71	45.39
Total PUFA	40.26	64.71	70.23	30.44
n-6 PUFA	35.85	63.23	27.30	25.40
n-3 PUFA	4.42	1.47	42.93	5.04
n-6/n-3 ratio	8.12	42.91	0.64	5.04
**Antioxidant compounds**				
Polyphenols (mg GAE/g)	7.95	90.42	15.33	90.72
Antioxidant capacity (mM Trolox/g)	24.57	496.0	19.87	118.50
Flavonoids (μg rutin/g) sample (μg RE/g)	4.51	100.08	1.35	120.01

*PUFA* polyunsaturated fatty acids, *DM* dry matter.

For the experiment, 120 Tetra SL LL laying hens, 50-week-old were used and assigned in a completely randomized design with three treatments (40 hens/group). A control diet (C) for laying hens based on corn and soybean meal with 2750 kcal/kg metabolizable energy and two supplemented diets, designed as follows: (T1) 9% rapeseed meal with 3% grapeseed meal, and (T2) 9% flaxseed meal with 3% sea-buckthorn meal were individually prepared by mixing the control diet (C) thoroughly with the designated supplements at the required incorporation levels as shown in Table 6. The dietary meals have been added to hens’ diets as rich sources of PUFA and antioxidants (Table 6). Each group was allocated to one of the three following dietary treatments: C, T1 and T2. The laying hens were housed in an experimental hall equipped with Big Dutchman double-sided, 3-tier battery cages dimensioned according to the sanitary-veterinary norms regarding the protection standards for handling of laying hens. Each cage was equipped with individual nipple drinker. The metal feed trough was divided to ensure that the hens were not able to consume feed assigned to the adjoining replicate. The layers had free access to water and feed which was administrated once daily at 08:30. The environmental conditions were controlled with a Viper Touch computer: 20–22 °C temperature, 60–65% humidity, 2.5–3% ventilation and a photoperiod of 16 h light with 8 h darkness cycle. The experiment was conducted on 42 days from 50 to 56 weeks of age, with 2 weeks of accommodation period, from 48 to 50 weeks of age.

**Table 6:** Ingredients and chemical composition of the diets.

Ingredients (%)	50–56 weeks of age		
	C	T1	T2
Corn	57.10	49.60	52.92
Soy meal	21.24	15.00	16.69
Sunflower meal	7.00	7.00	5.00
Rapeseed meal	0.00	9.00	0.00
Grapeseed meal	0.00	3.00	0.00
Sea-Buckthorn meal	0.00	0.00	3.00
Flaxseed meal	0.00	0.00	9.00
Sunflower vegetal oil	2.02	4.08	0.00
l-Lysine-HCl	0.06	0.08	0.14
dl-Methionine	0.10	0.06	0.19
Choline	0.05	0.05	0.05
Calcium carbonate	9.91	9.63	10.4
Phosphate	1.12	1.09	1.20
Mycotoxin inhibitor	0.05	0.05	0.05
Sodium chloride	0.35	0.36	0.36
Premix^a^	1.00	1.00	1.00
Total ingredients	100.00	100.00	100.00
**Chemical composition of the diets**			
Metabolizable energy (Kcal/kg)	2750.00	2750.00	2750.00
Crude protein (%)	16.50	16.50	16.50
Ether extract (%)	3.83	5.89	3.11
Crude fibre (%)	4.49	6.00	6.00
Palmitic acid (C16:0) (%)	10.17	9.19	13.53
Stearic acid (C18:0) (%)	2.56	2.62	2.38
Oleic acid (C18:1) (%)	28.68	29.00	24.48
α Linolenic acid (C18:3n3) (%)	0.78	1.22	15.41
Total PUFA (%)	57.77	58.61	58.72
n-6 PUFA (%)	56.20	54.78	44.84
n-3 PUFA (%)	1.57	3.83	13.88
n-6/n-3 ratio (%)	35.87	14.19	2.94
Polyphenols (mg GAE/g)	3.47	10.29	9.89
Antioxidant capacity (mM Trolox/g)	8.60	14.41	16.62
Flavonoids (μg rutin/g) sample (μg RE/g)	5.89	9.28	10.47

*C* control diet, *T1* control diet with 9% rapeseed meal and 3% grapeseed meal, *T2* control diet with 9% flaxseed meal and 3% sea-buckthorn meal, *PUFA* polyunsaturated fatty acids.

^a^The premix provided the following per kilogram of diet: vitamin A:13.500 IU; vitamin D3:3.000 IU; vitamin E:27 mg; vitamin K3: 2 mg; vitamin B1: 2 mg; vitamin B2: 4.8 mg; pantothenic acid: 14.85 mg; nicotinic acid: 27 mg; vitamin B6: 3 mg; vitamin B7: 0.04 mg; vitamin B9:1 mg; vitamin B12: 0.018 mg; vitamin C: 25 mg; manganese: 71.9 mg; iron: 60 mg; copper: 6 mg; zinc: 60 mg; cobalt: 0.5 mg; iodine: 1.14 mg; selenium: 0.18 mg.

### Proximate chemical composition analysis

The basic chemical composition analyses were determined on samples dried at 65 °C. Standardized methods were used to determine the nutrient concentration, performed according to the Regulation (CE) nr. 152/2009. Kjeldahl method was used for crude protein (CP) according to standard SR EN ISO 5983-2:2009 (Kjeltec 2300 Analyzer Unit, FOSS Analytical, Denmark). Crude fat (EE) was determined by extraction in organic solvents according to standard SR EN ISO 6492:2001 (Soxtec 2055—Foss Tecator, Sweden). For crude fiber (CF) the method with intermediary filtration was used according to standard SR EN ISO 6865:2002 (Fibertec 2010 System—Foss Tecator, Sweden).

### Laying performance and egg quality traits

During the 42 experimental days (6 weeks), egg production (%), average daily feed intake (g/day/layer), feed conversion ratio (g feed/g egg mass), laying percentage (%), and average egg weight (g/day), were monitored. Egg quality traits were determined as previously reported^55^. Briefly, by collecting 54 eggs (18 eggs/group) on last day of the trial, with homogenous weight and used to determine the physical quality parameters of the eggs: weight of the egg and its constituents (albumen, yolk, shell) with a Kern Precision Electronic Balance; Haugh Unit, an indicator of albumen quality was determined using an Egg Analyzer TM (ORKA Food Technology Ltd.), eggshell thickness, measured with an Egg Shell Thickness Gauge (Sanovo Engineering A/S, Denmark) and eggshell breaking strength, using an Egg Force Reader (Sanovo Engineering A/S, Denmark). The pH measurements (albumen and yolk) were performed with an INOLAB pH-meter (WTW, Weilheim, Germany). Yolk colour was determined by the Roche yolk colour fan (Hoffman-La Roche Ltd., Basel, Switzerland; colour scale from 15 dark orange, to 1, light pale). The fatty acid composition was determined from the pooled yolk samples, while the antioxidant capacity was determined separately from both yolk and white.

### Egg yolk fatty acids determination

The fatty acid (FA) profile from samples dried at 65 °C was determined using the fatty acid methyl ester (FAME) gas chromatography according to ISO/TS 17764-2 (2008), as described by Turcu et al.^12^. The FA from the total lipid extracts were converted to their methyl esters by trans esterification in methanol containing 3% concentrated sulfuric acid at 80 °C for 4 h. Methyl esters of FA were analysed in a Perkin Elmer-Clarus 500 chromatograph equipped with flame ionization detector (FID) and fitted with a BPX70 capillary column (60 m × 0.25 mm i.d., 0.25 μm film thickness). The column temperature was programmed at 5 °C/min, until 180–220 °C. The carrier gas was hydrogen (35 cm/s linear velocity at 180 °C) while the burning gas was air of analytical purity. The split ratio was 1:100. The injector and detector temperatures were 250 °C and 260 °C, respectively. FAME identification was done by comparison with retention times of the known standards. The results were expressed as mg of each FA per yolk. The average amount of each FA was used to calculate the sum of the total saturated (SFA), unsaturated (UFA), monounsaturated (MUFA) and polyunsaturated (PUFA) fatty acids.

### Calculation of health lipid indices

The health profile of enriched egg yolk were calculated based on the proportions of particular FA and their groups, as follows: saturation indices (SI), atherogenic indices (AI), thrombogenic indices (TI), peroxidability indices (PI), ratio of hypocholesterolemic and hypercholesterolemic (HH), hypercholesterolaemic saturated fatty acids (HFSA) oxidisability value (Cox), oxidative susceptibility (OS), desirable FA (DFA) and nutritive value indices (NVI) using the appropriate formulas^1,6,36,38^:

1 $${\text{SI}} = {{\left( {{\text{C}}14:0 + {\text{C}}16:0 + {\text{C}}18:0} \right)} \mathord{\left/ {\vphantom {{\left( {{\text{C}}14:0 + {\text{C}}16:0 + {\text{C}}18:0} \right)} {\left( {{\text{MUFA}} + {\text{PUFA}}} \right)}}} \right. \kern-\nulldelimiterspace} {\left( {{\text{MUFA}} + {\text{PUFA}}} \right)}}$$

2 $${\text{AI}} = {{\left( {4 \times {\text{C}}14:0 + {\text{C}}16:0 + {\text{C}}18:0} \right)} \mathord{\left/ {\vphantom {{\left( {4 \times {\text{C}}14:0 + {\text{C}}16:0 + {\text{C}}18:0} \right)} {\left( {{\text{MUFA}} + {\text{PUFA}}} \right)}}} \right. \kern-\nulldelimiterspace} {\left( {{\text{MUFA}} + {\text{PUFA}}} \right)}}$$

3 $${\text{TI}} = {{\left( {{\text{C}}14:0 + {\text{C}}16:0 + {\text{C}}18:0} \right)} \mathord{\left/ {\vphantom {{\left( {{\text{C}}14:0 + {\text{C}}16:0 + {\text{C}}18:0} \right)} {\left( {0.5 \times {\text{MUFA}} + 0.5 \times {\text{n}}6\;{\text{PUFA}} + 3 \times {\text{n}}3\;{\text{PUFA}} + {\text{n}}3:{\text{n}}6} \right)}}} \right. \kern-\nulldelimiterspace} {\left( {0.5 \times {\text{MUFA}} + 0.5 \times {\text{n}}6\;{\text{PUFA}} + 3 \times {\text{n}}3\;{\text{PUFA}} + {\text{n}}3:{\text{n}}6} \right)}}$$

4 $$\begin{aligned} {\text{PI}} & = \left( {\% \;{\text{monoenoic}}\;{\text{FA}} \times 0.025} \right) + \left( {\% \;{\text{dienoic}}\;{\text{FA}} \times 1} \right) + \left( {\% \;{\text{trienoic}}\;{\text{FA}} \times 2} \right) \\ & \quad + \left( {\% \;{\text{tetraenoic}}\;{\text{FA}} \times 4} \right) + \left( {\% \;{\text{pentaenoic}}\;{\text{FA}} \times 6} \right) + \left( {\% \;{\text{hexaenoic}}\;{\text{FA}} \times 8} \right) \\ \end{aligned}$$

5 $$\begin{aligned} {\text{HH}} & = ({\text{C}}18:1{\text{n}}9 + {\text{C}}18:2{\text{n}}6 + {\text{C}}20:4{\text{n}}6 + {\text{C}}18:3{\text{n}}3 + {\text{C}}20:5{\text{n}}3 + {\text{C}}22:5{\text{n}} - 3 \\ & \quad {{ + {\text{C}}22:6{\text{n}}6)} \mathord{\left/ {\vphantom {{ + {\text{C}}22:6{\text{n}}6)} {\left( {{\text{C}}14:0 + {\text{C}}16:0} \right)}}} \right. \kern-\nulldelimiterspace} {\left( {{\text{C}}14:0 + {\text{C}}16:0} \right)}} \\ \end{aligned}$$

6 $${\text{HSFA}} = \left( {{\text{C}}14:0 + {\text{C}}16:0} \right)$$

7 $${\text{Cox}} = {{\left( {{\text{C}}18:1 + 10.3 \times {\text{C}}18:2 + 21.6 \times {\text{C}}18:3} \right)} \mathord{\left/ {\vphantom {{\left( {{\text{C}}18:1 + 10.3 \times {\text{C}}18:2 + 21.6 \times {\text{C}}18:3} \right)} {100}}} \right. \kern-\nulldelimiterspace} {100}}$$

8 $${\text{OS}} = {\text{MUFA}} + 45 \times {\text{C}}18:2 + 100 \times {\text{C}}18:3$$

9 $${\text{DFA}} = \left( {{\text{C}}18:0 + {\text{UFA}}} \right)$$

10 $${\text{NVI}} = {{\left( {{\text{C}}18:0 + {\text{C}}18:1} \right)} \mathord{\left/ {\vphantom {{\left( {{\text{C}}18:0 + {\text{C}}18:1} \right)} {{\text{C}}16:0}}} \right. \kern-\nulldelimiterspace} {{\text{C}}16:0}}$$

The peroxidability indices of the lipid, were calculated from the concentrations of specific FA and were quantified based on their abundance (%, w/w). The total monoenoic FA were given by the miristic, pentadecanoic, palmitic, heptadecanoic and stearic multiplied by 0.025. The dienoic FA were given by linoleic and eicosadienoic multiplied by 1. Trienoic fatty acids which are formed from all 18-carbon chain with three cis double links, linolenic γ, α-linolenic, eicosatrienoic (C20: 3n6) and eicosatrienoic (C20:3n3) multiplied by 2, while tetraenoic FA were calculated from arachidonic acid (C20:4n6) and docosatetraenoic (C22:4n6), which are formed from all 20-carbon chain and four cis double links multiplied by 6. For pentaenoic we determined only docosapentaenoic (C22:5n3) multiplied by 6 and for hexaenoic we determined docosahexaenoic acid (C22:6n3) multiplied by 8.

### Determination of antioxidant compounds

The total polyphenol concentration (TPC) and total antioxidant capacity (TAC) were determined as described by Olteanu et al.^56^. Briefly, the TPC were determined spectrophotometrically in the methanolic extracts of samples, using a UV–Vis Thermo Scientific spectrophotometer, and the results were expressed in mg equivalents gallic acid/g (mg GAE/g sample). The TAC of the methanol extracts was determined by using the DPPH method, with an UV–Vis Analytik Jena Specord 250 Plus spectrophotometer with thermostatic carousel. The obtained results of TAC are expressed in Trolox equivalents/g sample (mM Trolox/g sample).

### Statistical analysis

The statistical model was applied as reported previously^55^. One-way analysis of variance (ANOVA), using STATVIEW for WINDOWS (SAS, version 6.0) was carried out to determine the effect of diets on egg quality parameters, fatty acid composition, total polyphenols and antioxidant content in eggs. Significance between individual mean was identified using the Tukey’s multiple range tests. Mean differences were considered significant at *p* < 0.05.

The Principal Component Analysis (PCA) was obtained from the corresponding function of MATLAB & SIMULINK software package, used to reveal the correlation structure between the investigated parameters. PCA is a multivariate technique commonly adopted to reduce the dimensionality of data. By reducing the number of variables, the use of PCA allows an easier analysis and comparison of eggs quality characteristics and similarities between the groups. To obtain the PCA representation we considered centred and normalized version of the data. The first component (PC1) covered 75% of the global variance of the data while the second component (PC2) covered about 20% of the global variance.

## Data Availability

The datasets during and/or analysed during the current study are available from the corresponding authors on reasonable request.
